# Isorhynchophylline inhibits inflammatory responses in endothelial cells and macrophages through the NF-κB/NLRP3 signaling pathway

**DOI:** 10.1186/s12906-023-03902-3

**Published:** 2023-03-11

**Authors:** Li-Hua Wang, Zheng-Wei Gu, Jie Li, Wen-Qing Yang, Yun-Lun Li, Dong-Mei Qi, Dan-Yang Wang, Hai-Qiang Jiang

**Affiliations:** 1 Innovative Institute of Chinese Medicine and Pharmacy, Shandong University of Traditional Chinese Medicine, Jinan, 250000 China; 2 Shandong University of Traditional Chinese Medicine, Jinan, 250000 China

**Keywords:** Inflammation, NF-κB, NLRP3, Human umbilical vein endothelial cells, Isorhynchophylline

## Abstract

**Background:**

Atherosclerosis is a chronic inflammatory disease of arterial wall, which is closely related to inflammatory reaction. In this study, the anti-inflammatory effect of isorhynchophylline was studied by NF- κB / NLRP3 pathway.

**Methods:**

(1) ApoE^−/−^ mice were fed with high-fat diet to establish atherosclerotic model, while C57 with the same genetic background was fed with common diet as control group. Body weight was recorded and blood lipids were detected. The expression of NLRP3, NF-κB, IL-18 and Caspase-1 in aorta was detected by Western-Blot and PCR, and plaque formation was detected by HE and oil red O staining. (2) Lipopolysaccharide interfered with Human Umbilical Vein Endothelial Cells (HUVECs) and RAW264.7 to form inflammatory model, and was treated with isorhynchophylline. The expression of NLRP3, NF-κB, IL-18 and Caspase-1 in aorta was detected by Western-Blot and PCR, and the ability of cell migration was detected by Transwell and scratch test.

**Results:**

(1) the expression of NLRP3, NF- κB, IL-18 and Caspase-1 in aorta of model group was higher than that of control group, and plaque formation was obvious. (2) the expressions of NLRP3, NF- κB, IL-18 and Caspase-1 in HUVECs and RAW264.7 model groups were higher than those in control group, while isorhynchophylline decreased their expression and enhanced cell migration ability.

**Conclusion:**

Isorhynchophylline can reduce the inflammatory reaction induced by lipopolysaccharide and promote the ability of cell migration.

**Supplementary Information:**

The online version contains supplementary material available at 10.1186/s12906-023-03902-3.

## Introduction

Atherosclerosis (AS), as the main pathological basis of cardiovascular disease, is a chronic and diffuse vascular endothelial inflammatory response. Typical triggers of inflammation are infection and tissue damage [1]. IL-18 ^−/−^ × ApoE ^−/−^ mice showed a 50% increase in serum cholesterol, but a reduction in the size of atherosclerotic lesions, providing strong evidence for the inflammatory hypothesis of atherosclerotic thrombosis [2].

Arterial inflammation is triggered by damage to the endothelium, usually at arterial branch points or areas where blood flow is obstructed, resulting in endothelial cell activation and recruitment of inflammatory cells to the vessel wall. At the site of endothelial activation, the structure changes, and the exposure of proteoglycans promotes the retention of low-density lipoprotein particles in the intima, and foam cells are generated as macrophages gradually absorb the modified lipoproteins. Foam cells and lipids form the lipid core of the plaque, and macrophages in the lesion produce large amounts of metalloproteinases that promote plaque instability and rupture around the shoulder to form “vulnerable plaques” [3, 4].

Endothelial cells metabolize actively, regulate vascular tension, inflammatory response and generate new blood vessels, and maintain vascular homeostasis through synthesis and secretion and paracrine of metabolites [5, 6]. Long-term exposure to cardiovascular hazards or harmful circulation can disrupt endothelial defense mechanisms, causing endothelial cell damage. Endothelial cell injury is an early step in the beginning of AS, and endothelial cell permeability increases after injury, which is more conducive to the transmission of lipoprotein and inflammatory cells, thus promoting the development of AS [7, 8].

In the injured site of atherosclerotic vascular endothelium, macrophages recruit immune cells and participate in inflammatory response by producing pro-inflammatory factors and chemokines. Lipoprotein is oxidized and deposited in the arterial wall to promote macrophage foaming and participate in the formation of plaques. The expression of pro-inflammatory factors by macrophages in plaques can accelerate the progression of AS [3]. Macrophages are plastic, so macrophages have the ability to promote inflammation and inhibit inflammation [9]. Macrophages exist at different sites in the plaque and the affected macrophages polarize and activate the phenotype [10, 11]. The typical inflammatory macrophage phenotype is M1, which is usually induced by incubation with interferon-γ (IFN-γ) and toll-like receptor 4 (TLR4) ligand lipopolysaccharide. It is a mature differentiation of monocytes that phagocytize excess lipids to form foam cells, while producing large amounts of inflammatory mediators [11]. Several subpopulations of alternatively activated macrophage populations, called M2 macrophages. The M2 phenotype can be induced by incubation of macrophages with IL-4 and IL-13, which have anti-inflammatory effects [12]. Although the existing M2 phenotype is less sensitive to lipids, when the plaque continues to develop, the M2 phenotype will be transformed into m1-phenotype macrophages, promoting the continuous occurrence of inflammatory response [13]. During the progression of atherosclerosis, the migration of macrophages from atherosclerotic plaques decreases, thus maintaining the inflammatory state of plaques and leading to the progression of chronic and more complex lesions. Macrophages are usually removed from the site where inflammation is resolved by lymphatic migration to the lymph nodes [14], thereby reducing intravascular inflammation.

Vascular wall inflammation plays an important role in the process of AS lesions, and NLRP3 inflammasomes, as the core part of the inflammatory response, mediate the occurrence and development of AS [15]. NOD-like receptor pyrin domain containing 3 (NLRP3) inflammasome is composed of NLRP3, apoptosis-associated speck-like protein containing (ASC) and cysteine-containing Acid aspartate proteolytic enzyme 1 (cysteinyl aspartate specific proteinase-1, Caspase-1) composition. Activated NLRP3 inflammasome cleaves Caspase-1, releases mature interleukin-18 and other pro-inflammatory factors, triggering vascular inflammatory response [16]. Experiments have shown that the expression of IL-18 in the serum of Caspase-1 knockout mice is reduced, and the recruitment of monocytes is weakened [17]. The nuclear factor NF-κB pathway has long been recognized as a typical pro-inflammatory signaling pathway, mainly based on the role of NF-κB in the expression of pro-inflammatory genes, including cytokines, chemokines, and adhesion molecules. Similar studies have shown that the production of proinflammatory cytokines in human atherosclerotic plaques is also dependent on NF-κB [18]. Studies have shown that NF-κB-induced NLRP3 expression is sufficient for NLRP3 to mediate inflammasome formation, suggesting that blocking the functional crosstalk that occurs between NF-κB and NLRP3 may reduce inflammation [19].

Isorhynchophylline (IRN) is a common bioactive ingredient extracted from rubiaceae uncaria. It is a tetracyclic indole oxide alkaloid with the molecular formula C_22_H_26_N_2_O_4_ (384.47 g/mol), and has many biological effects such as anti-hypertension, anti-proliferation, anti-inflammation and neuroprotection [20–23]. For example, IRN has been reported to reduce LPS-induced production of inflammatory cytokines in mouse microglia [24]. Other study has found that IRN has anti-inflammatory and antioxidant effects on endotoxin-stimulated mouse alveolar macrophages [25]. However, whether IRN can play a potential anti-inflammatory role in endothelial cells and macrophages remains unknown.

The purpose of this study was to explore the inhibitory effect of isorhynchophylline on lipopolysaccharide-induced inflammatory responses of HUVECs and RAW264.7.

## Materials and methods

### Antibodies

NF-κB p65 (15101S, Cell Signaling Technology, USA), NLRP3 (8242S, Cell Signaling Technology, USA), Caspase-1 (3866S, Cell Signaling Technology, USA), IL-18 (12242S, Cell Signaling Technology, USA), β-actin (Abways Technology, Shanghai), HRP-linked Anti-Rabbit IgG (H + L) (7074S, Cell Signaling Technology, USA),Goat Anti-Mouse IgG (H + L) (SparkJade, China).

### Animals

Blank control group: female and male half, 12 mice; AS model group: female and male half, 12 mice (Animals were purchased from Charles River (Weitong Lihua) Laboratory Animal Technology Co. Ltd. (Beijing, China).). 8-week-old ApoE^−/−^ mice were fed with high-fat diet with fat content of 21% and cholesterol content of 0.25% for 12 weeks, while 8-week-old C57BL/6 mice with the same genetic background were fed with normal diet as control group. During the feeding of mice, body weight, hair color, survival rate and other growth states of mice were recorded. After 12 hours of fasting and water deprivation, the mice were anesthetized by intraperitoneal injection of sodium pentobarbital (50 mg/kg) (China, Beijing Chemical Reagent Co., 061206), blood samples were collected from orbit for blood lipid analysis, and the mice were killed after neck removal. Normal saline was injected through the left ventricular puncture vascular system, and the aorta and other tissues were collected. The protocol of animal experiments was approved by the Ethics Committee of Shandong University of Traditional Chinese Medicine. The Protocol permission number is SDUTCM20201030002. Experimental Animal Production License No.: SCXK (Beijing) 2016–0011. Animal care and procedures comply with the ethical guidelines issued by the International Scientific Committee on Experimental Animals (ICLAS).

### Oil red O staining

The mouse aorta was dissected in cold PBS. 4% paraformaldehyde was fixed at 4 °C for 16 h, rinsed with water for 10 minutes, and then rinsed with 60% isopropanol. Dye the blood vessels with oil red O for 15 minutes, shake gently, rinse with 60% isopropanol, and then rinse with water 3 times. The heart samples were collected and the cross section of the aortic root was made. The lesion site was identified by oil red O and the image was observed under microscope.

### HE staining

The aortic roots of each group were fixed with 10% formalin, embedded in paraffin, sliced into 5 μm, stained with HE, and observed under stereo fluorescence microscope.

### Cell viability assay

Cell viability was detected by cell counting kit (CCK8, MedChemExpress, New Jersey, USA). Human umbilical vein endothelial cells were cultured in 96-well plate at the ratio of 5 × 10^3^ cells per well. RAW264.7 was cultured in 96-well plate with 1 × 10^4^ cells per well. After 24 hours, the culture medium was changed and the cells were stimulated with different concentrations of LPS. Then, the cells were cultured in 37 °C and 5% CO_2_ humidified incubator for different times, and then cultured in CCK-8 for 1 h.

### Quantitative real-time PCR

RNA was extracted by using TRIzol Reagent (Invitrogen, CA, USA) according to the manufacturer’s protocol. cDNA was synthesized using SPARKscript IIRT Plus Kit (SparkJade, China). The qPCR analysis with specific primer pairs was performed with 2 × SYBR Green qPCR Mix (SparkJade, China). The gene expression level was calculated by 2^-ΔΔCt^ method and normalized to β-actin mRNA expression level. The primer (Biosune, Shanghai, China) base sequence is shown in Table 1.

**Table 1:** Primer Sequences

Gene	Forward Primer (5′-3′)	Reverse Primer (5′-3′)
Human IL-18	ATTGACCAAGGAAATCGGCCTC	GGTCCGGGGTGCATTATCTCT
Human Caspase-1	TTGGAGACATCCCACAATGG	TGAAAATCGAACCTTGCGGA
Human NF-kB p65	AAGAAGAGTCCTTTCAGCGGACC	TGCGGGAAGGCACAGCAAT
Human NLRP3	GAGCCGAAGTGGGGTTCAGA	CTTCAATGCTGTCTTCCTGGC
Human b-actin	CATGTACGTTGCTATCCAGGC	CTCCTTAATGTCACGCACGAT
Mouse IL-18	TCAAAGTGCCAGTGAACCCC	GGTCACAGCCAGTCCTCTTAC
Mouse Caspase-1	CGTACACGTCTTGCCCTCAT	AACTTGAGCTCCAACCCTCG
Mouse NF-kB p65	ATCGCCACCGGATTGAAGAG	CGGGGTTCAGTTGGTCCATT
Mouse NLRP3	TCTGCACCCGGACTGTAAAC	CATTGTTGCCCAGGTTCAGC
Mouse b-actin	GGCTGTATTCCCCTCCATCG	CCAGTTGGTAACAATGCCATGT

### Western blot analysis

Cells or tissues were homogenized in cold RIPA lysis buffer supplemented with phenylmethanesulfonyl fluoride (PMSF). Protein concentrations were measured using a BCA protein assay kit (Dalian Meilun Biotechnology, Dalian, China). Protein samples were separated by sodium dodecyl sulfate-polyacrylamide gel electrophoresis (SDS-PAGE) and then transferred to polyvinylidene difluoride (PVDF) membranes (Merkck, Germany) by electroblotting. After sealing the film with 5% skim milk for 1 hour, the membranes were incubated with primary antibodies (1:1000) and β-actin (1:5000) overnight at 4 °C, and then incubated with HRP-linked Anti-Rabbit IgG (H + L) (Cell Signaling Technology, USA) (1:3000) and Goat Anti-Mouse IgG (H + L) (SparkJade, China) (1:5000). After 1 h, park ECL Super (SparkJade, China) was used to detect the bands, and the average densitometric analysis was conducted using ImageJ software.

### Cell culture and drug treatment

Human umbilical vein endothelial cells (HUVECs, OTWO Biotech, HTX2104) were cultured in ECM containing 25 mL fetal bovine serum (FBS), 5 mL endothelial cell growth supplement, and 5 mL antibiotic solution. Cells were maintained at 37 °C and 5% CO_2_ in a humidified incubator. The control group were cultured only with DMEM, the model group were incubated with DMEM+ 10 μg/mL of LPS (SIGMA). The treat group was cultured with DMEM+ 10 μg/mL of LPS and 12 mg/L of IRN (Shanghai Yuanye Bio-Technology Co. Ltd., 6859-01-4). All cells were incubated for an additional 24 h before analysis.

RAW264.7 (Meilunbio, PWE-MU004–2) were cultured in DMEM containing 10% FBS and 1% antibiotic solution. Cells were maintained at 37 °C and 5% CO_2_ in a humidified incubator. The control group were cultured only with DMEM, the model group were incubated with DMEM+ 10 μg/mL of LPS. The treat group was cultured with DMEM+ 10 μg/mL of LPS and 12 mg/L of IRN. All cells were incubated for an additional 24 h before analysis.

### Scratch assay and transwell assay

Cells were grown in a humidified incubator at 37 °C and 5% CO_2_ for 24 h. Ricks were generated in the cell monolayer using a 10 μL straw tip. Imaging was performed at 0/3/6/9/12 h. Cell migration ability was determined by the transwell assay.

### Statistical analysis

All experimental data were expressed as means ± standard error of mean (SEM). Two-group comparison was performed using a t-test for independent samples. Raw data were analyzed with SPSS 25.0 software and images were processed with GraphPad Prism 7. *P*-Values< 0.05 were considered statistically significant. Each experiment was repeated three times.

## Results

### Inflammatory genes are highly expressed in animal models

**Fig. 1:**
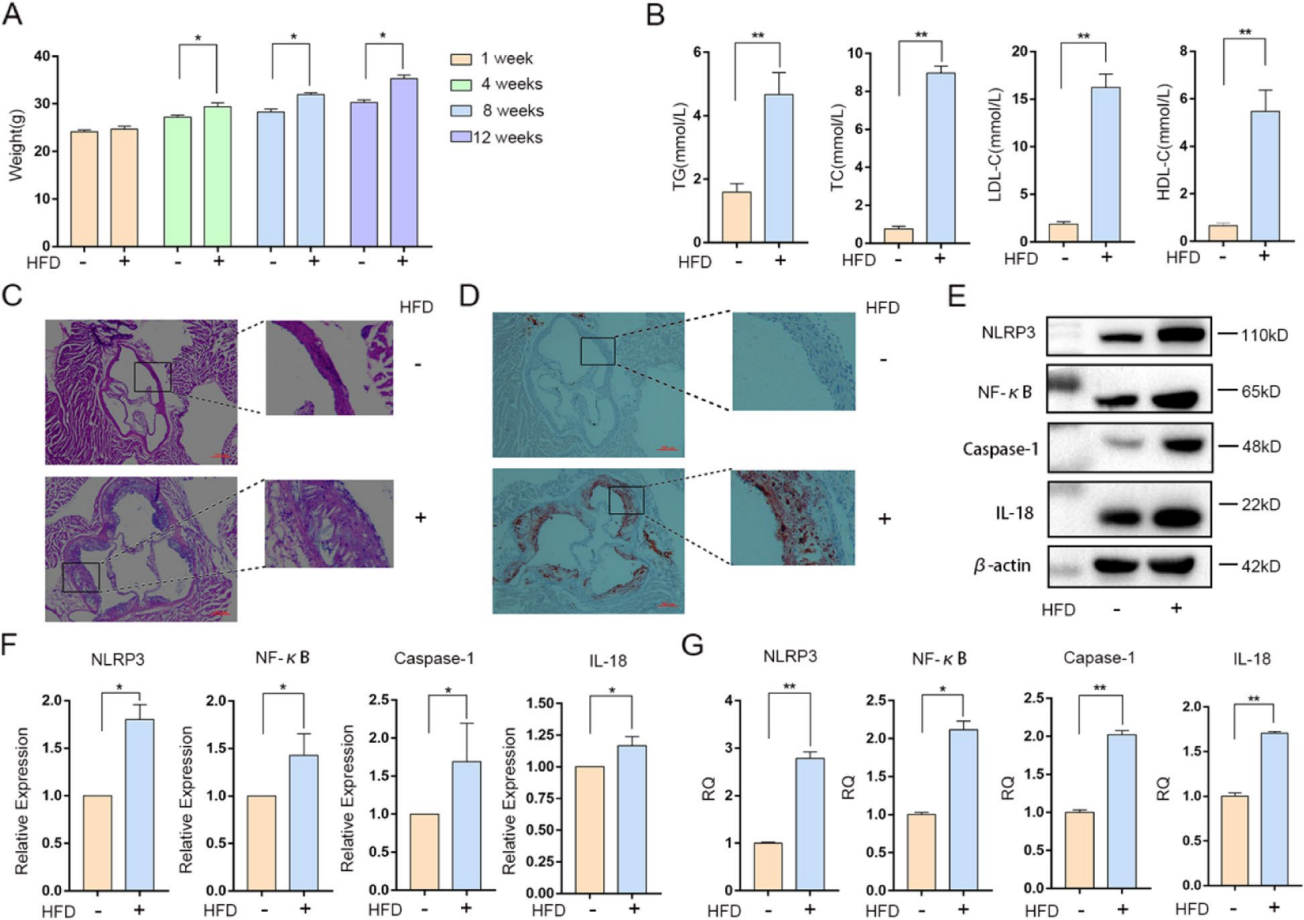
Evaluation the model of AS induced by high fat diet. **A** Body weight of mice at 1, 4, 8 and 12 weeks. *P<0.05, ** *P*<0.01. **B** Plasma total cholesterol (TC), triglyceride (TG), HDL cholesterol (HDL-C), LDL cholesterol (LDL-C), ox-LDL levels (*n* = 12). *P<0.05, ** *P*<0.01. **C** HE staining of aortic root. Scale bar, 200 μm. The picture on the right is four times larger than the one on the left. **D** Frozen sections of aortic root were stained with Oil Red O. Scale bar, 200 μm. The picture on the right is four times larger than the one on the left. **E**-**F** The expression of NLRP3, NF-кB, Caspase-1 and IL-18 were detected by Western Blot (*n* = 3). *P<0.05, ** *P*<0.01. **G** The expression levels of NLRP3, NF-кB, Caspase-1 and IL-18 were detected by RT-PCR (*n* = 3). *P<0.05, ** *P*<0.01

In order to verify the abnormal expression of inflammatory factors in AS, we constructed the AS mouse model. We regularly documented the weight every 4 weeks (Fig. 1A). At 4 weeks the model group was about 1.08 times as much as the normal group, at 8 weeks the model group was about 1.13 times as much as the normal group and at 12 weeks the model group was about 1.17 times as much as the normal group. After 12 weeks of HFD, plaque formation increased significantly in model group, accompanied by increased levels of TG, TC, LDL-C and HDL-C (Fig. 1B).TG content in the model group was three times that in the normal group. The content of TC in model group was 12 times of that in normal group. The level of LDL-C in the model group was nine times higher than that in the normal group and the HDL-C content of the model group was 7 times higher than that of the normal group. Aortic oil red O staining and HE staining showed that plaque formation and lipid accumulation were significantly increased in the model group (Fig. 1C-D). The protein expression levels of NLRP3, NF-кB, Caspase-1 and inflammatory factor IL-18 in the model group were all higher than those in the control group (Fig. 1E-F). Besides, the expression of NLRP3, NF-кB, Caspase-1 and the inflammatory factor IL-18 were measured in the aorta. PCR results showed that the expression of NLRP3 and NF-кB was 2.79 times and 2.12 times that of the control group. The Caspase-1 model group was 1.73 times higher than the control group and the IL-18 model group was 1.52 times higher than the control group (Fig. 1G).

### IRN suppressed inflammation by inhibiting the NF-кB /NLRP3 pathway in HUVECs

**Fig. 2:**
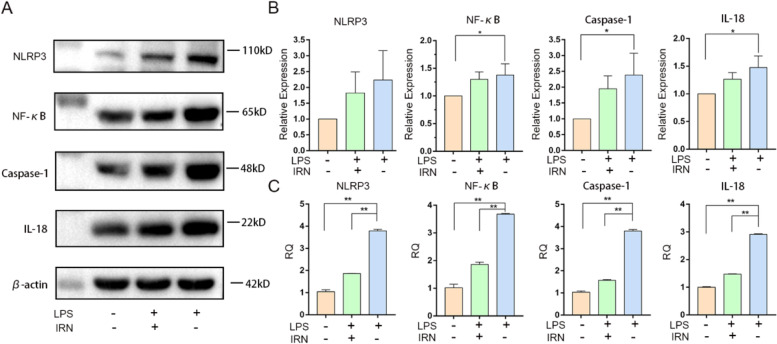
Effects of IRN on inflammatory response of NF-кB /NLRP3 signaling pathway in HUVECs. **A**-**B** The expression of NLRP3, NF-кB, Caspase-1 and IL-18 in the pathway were detected by Western Blot (*n* = 3). *P<0.05, ** *P*<0.01. **C** The expression levels of NLRP3, NF-ΚB, Caspase-1 and IL-18 were detected by RT-PCR (*n* = 3). *P<0.05, ** *P*<0.01

To investigate the anti-inflammatory effect of IRN and examine whether IRN inhibit NF-кB /NLRP3 activity in atherosclerosis, we detected the expression of inflammation related factors in LPS-induced HUVECs. Western blot results showed that the expression of NLRP3, NF-кB, Caspase-1 and inflammatory factor IL-18 in LPS-induced model cells was higher than that in control cells, and IRN significantly inhibited the expression of NLRP3, NF-кB, Caspase-1 and inflammatory factor IL-18 in LPS-induced model cells (Fig. 2A-B). PCR results showed that the expression of NLRP3 model group was 4.27 times, NF-кB model group was 4.49 times, Caspase-1 model group was 3.82 times, IL-18 model group was 2.87 times than that of control group. After administration, the expressions of NLRP3, NF-кB, Caspase-1 and IL-18 decreased (Fig. 2C).

### IRN suppressed inflammation by inhibiting the NF-кB /NLRP3 pathway in RAW264.7

**Fig. 3:**
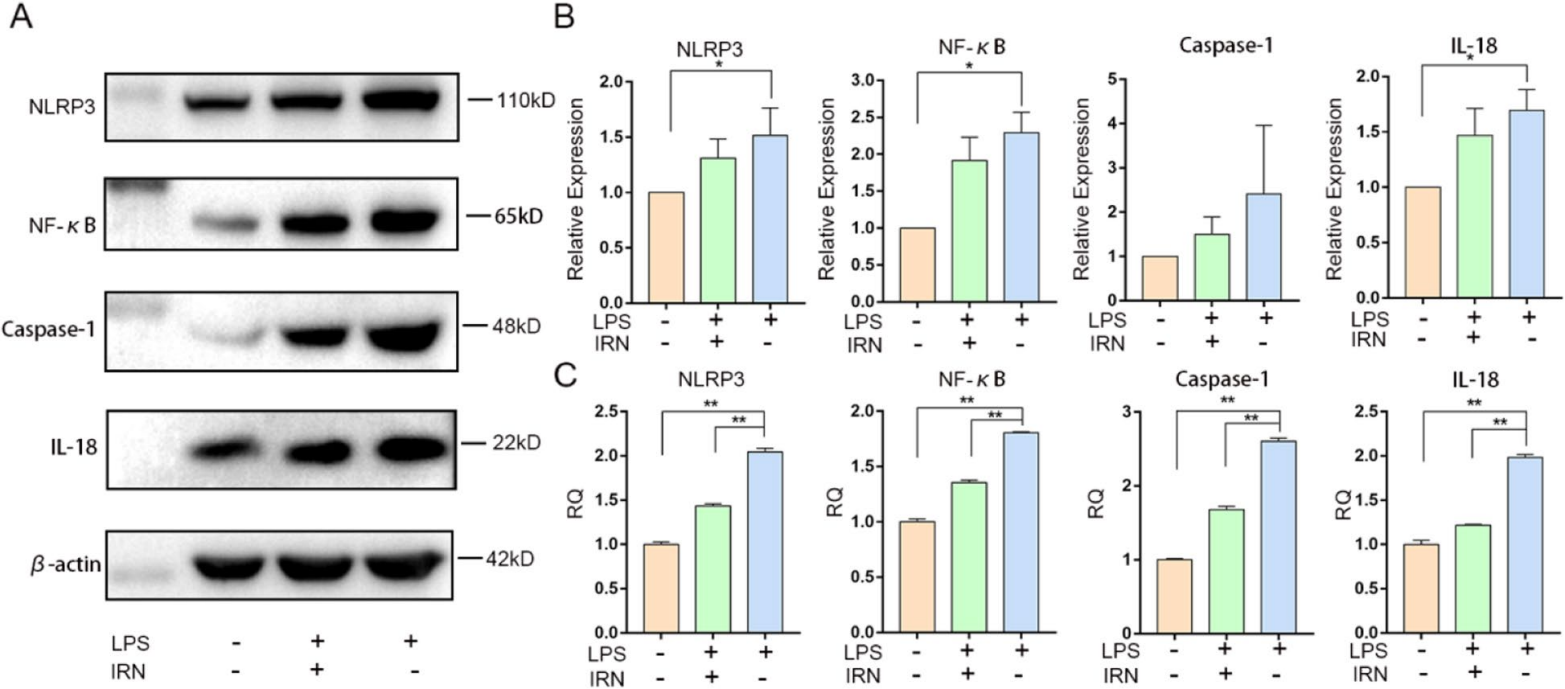
Effects of IRN on inflammatory response of NF-кB /NLRP3 signaling pathway in RAW264.7. **A**-**B** The expression of NLRP3, NF-кB, Caspase-1 and IL-18 in the pathway were detected by Western Blot (*n* = 3). *P<0.05, ** *P*<0.01. **C** The expression levels of NLRP3, NF-кB, Caspase-1 and IL-18 were detected by RT-PCR (*n* = 3). *P<0.05, ** *P*<0.01

To investigate the anti-inflammatory effect of IRN and examine whether it inhibits NF-кB /NLRP3 activity in atherosclerosis, we tested the expression of inflammation related factors in RAW264.7 induced by LPS. Western blot results showed that the expression of NLRP3, NF-кB, Caspase-1 and inflammatory factor IL-18 in LPS-induced model cells was higher than that in control cells, and IRN significantly inhibited the expression of NLRP3, NF-кB, Caspase-1 and inflammatory factor IL-18 in LPS-induced model cells (Fig. 3A-B). PCR results showed that the expression of NLRP3, NF-кB, Caspase-1 and IL-18 was 2.95 times higher than that of the control group, 2.54 times higher than that of the control group, 1.71 times higher than that of the control group, 2.72 times higher than that of the control group. After administration, the expressions of NLRP3, NF-кB, Caspase-1 and IL-18 decreased (Fig. 3C).

### The effect of IRN on migration of HUVECs was evaluated by scratch test and transwell test

**Fig. 4:**
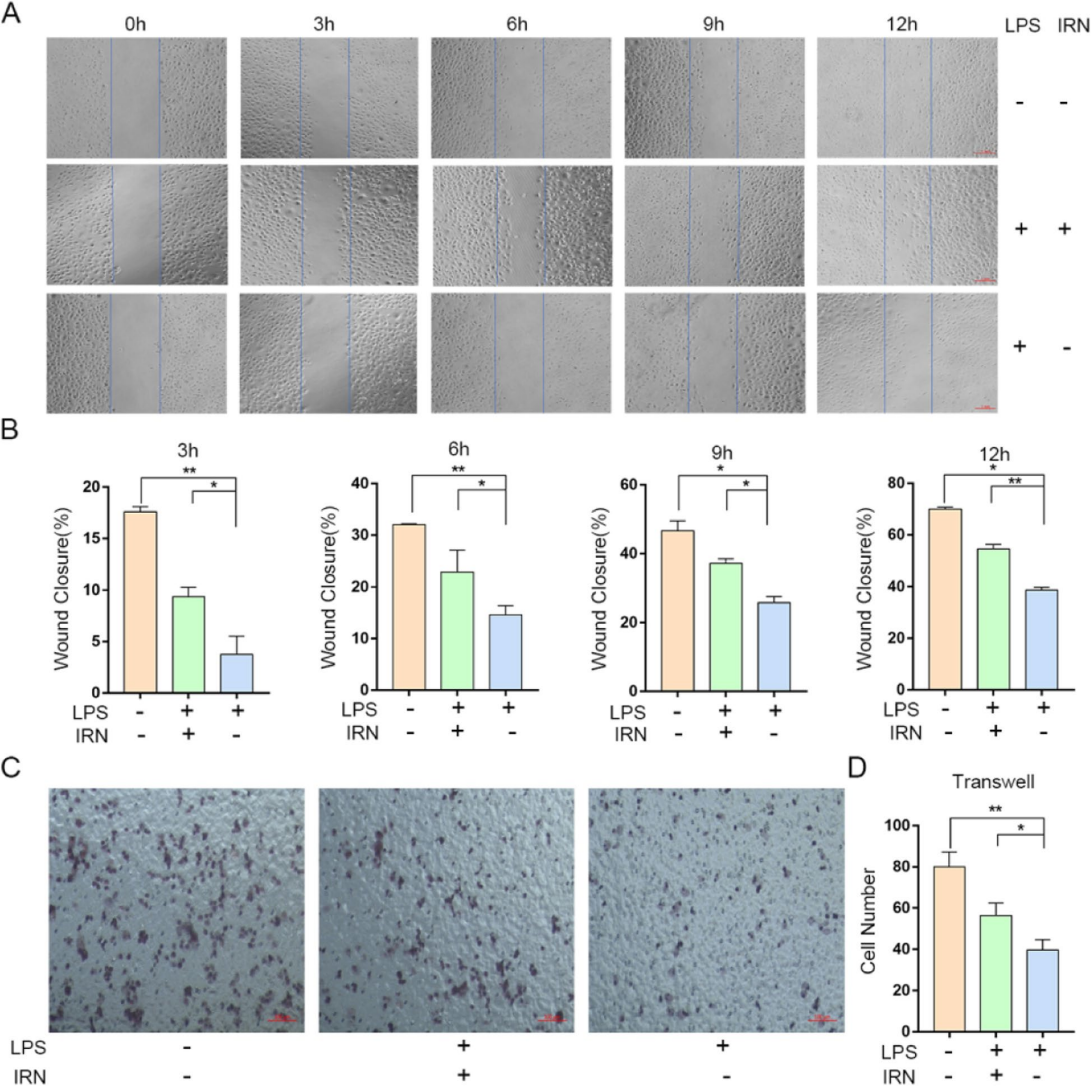
Migration capacity of HUVECs treated by IRN. **A** Representative micrographs of scratch analysis at different times in the experiment. Scale bar, 1 mm. **B** Wound closure represented as migration ability in three groups (*n* = 3). *P<0.05, ** *P*< 0.01. **C** HUVECs migration was determined by transwell assay. Scale bar, 100 μm. **D** Statistical data of migration cells was shown (*n* = 3). *P<0.05, ** *P*<0.01

Compared with 0 h, at 3, 6, 9 and 12 h, all groups had different numbers of cells crossing the blue line. The total number of cells crossing the blue line in the model group at 3, 6, 9 and 12 hours was lower than that in the control group (Fig. 4A-B). However, compared with the model group, the number of HUVECs processed with IRN increased at different time points (Fig. 4A-B). The migration ability of HUVECs was determined by Transwell method, and the results were consistent with those of scratch method. After 6 hours of culture, the migration ability of endothelial cells in the endotoxin treatment group was significantly lower than that in the control group. In addition, the number of HUVECs in the treatment group was significantly higher than that in the model group (Fig. 4C-D). As mentioned above, from the overall trend, the migration ability of HUVECs is obviously impaired by LPS, while IRN can inhibit the impaired of LPS.

### The effect of IRN on migration of RAW264.7 was evaluated by scratch test and transwell test

**Fig. 5:**
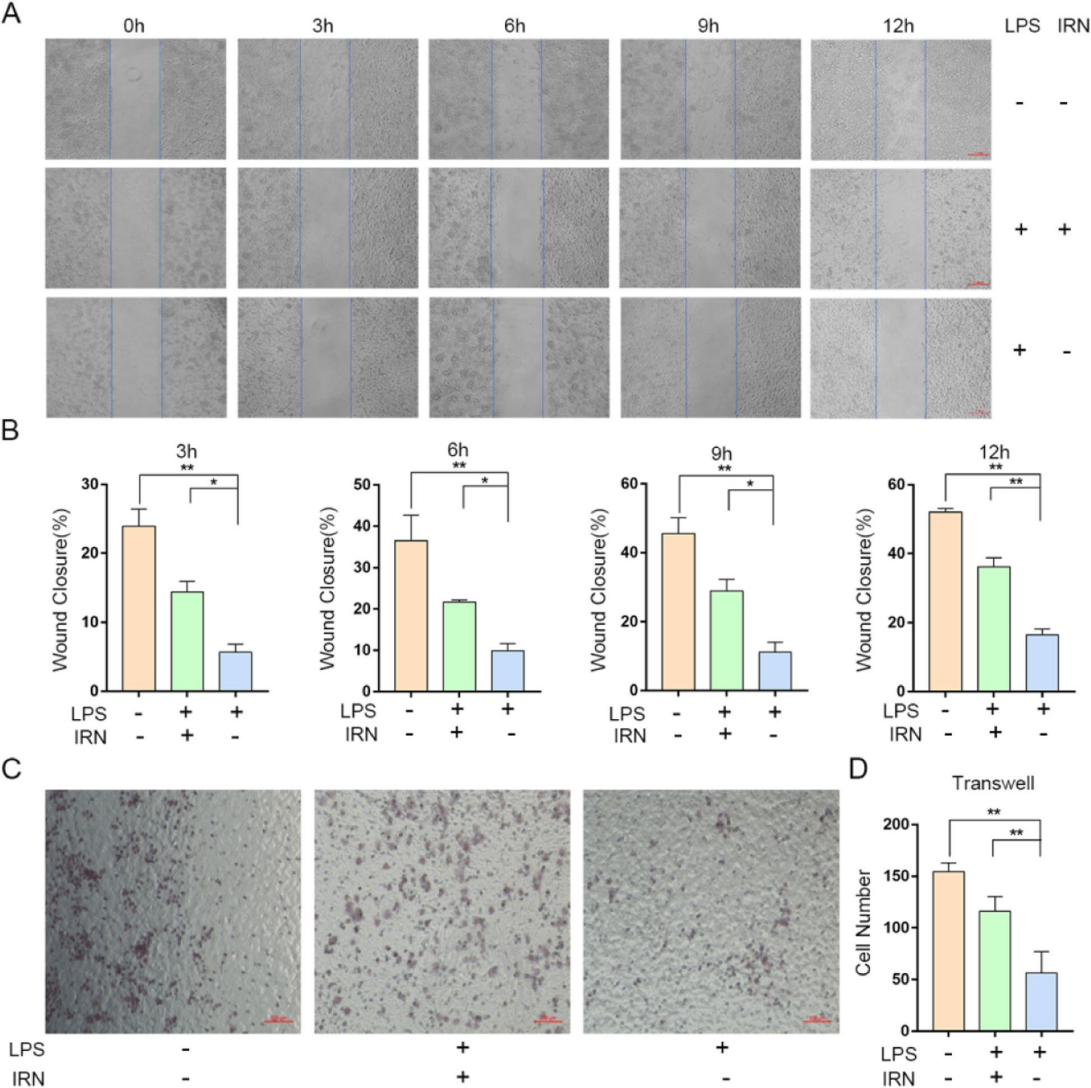
Migration capacity of RAW264.7 treated by IRN. **A** Representative micrographs of scratch analysis at different times in the experiment. Scale bar, 1 mm. **B** Wound closure represented as migration ability in three groups (*n* = 3). *P<0.05, ** *P*< 0.01. **C** RAW264.7 migration was determined by transwell assay. Scale bar, 100 μm. **D** Statistical data of migration cells was shown (*n* = 3). *P<0.05, ** *P*<0.01

Compared with 0 h, at 3, 6, 9 and 12 h, all groups had different numbers of cells crossing the blue line. The total number of cells crossed the blue line at 3, 6, 9 and 12 h in the model group was lower than that in the control group (Fig. 5A-B). However, compared with the model group, the number of RAW264.7 treated with IRN increased at different time points (Fig. 5A-B). The migration ability of RAW264.7 was determined by Transwell method, and the results were in good agreement with those of scratch test. After 6 hours of culture, the migration ability of RAW264.7 treated with endotoxin was significantly lower than that of the control group. In addition, the number of RAW264.7 in the treatment group was significantly higher than that in the model group (Fig. 5C-D). As mentioned above, from the overall trend, the migration ability of RAW264.7 is obviously impaired by LPS, while IRN can inhibit the impaired of LPS.

## Discussion

AS is a chronic vascular wall inflammatory disease [4], and inflammation has been shown to play an important role in the occurrence and development of atherosclerotic plaques [26]. The inflammatory reaction of arterial wall runs through the whole process of initiation, progression and plaque rupture and thrombosis of AS [27]. Dyslipidemia, especially hypercholesterolemia, which is mainly manifested as increased low-density lipoprotein (LDL-C) and total cholesterol (TC), is one of the main risk factors for atherosclerotic vascular disease. Low density lipoprotein (LDL-C) and high-density lipoprotein cholesterol (HDL-C) are currently the two most studied lipoproteins in atherosclerosis, and triglyceride (TG) levels also play a crucial role [28, 29]. After the vessel wall is damaged, endothelial cells, macrophages, plaques, etc. will release pro-inflammatory factors to maintain and enhance local inflammation and the development of atherosclerotic lesions [30]. In this study, we successfully established an AS mouse model by high-fat chow feeding, and ApoE^−/−^ mice had elevated serum levels of TG, TC, and LDL-C, but also elevated HDL-C (a protective lipoprotein). This may be due to ApoE being knocked out, which is detrimental to cholesterol excretion and allows a compensatory increase in HDL-C [31]. There was also a significant increase in aortic arch plaques in the model group (Fig. 1). The expressions of NLRP3, NF-κB, Caspas-1 and IL-18 were significantly increased in ApoE^−/−^ mice fed with high fat diet (Fig. 1). In conclusion, the above animal experimental results suggest that there is an inflammatory response in AS, which may be related to NF-κB/NLRP3 pathway.

NLRP3 is the most representative inflammasome involved in the inflammatory response of AS [32]. NLRP3 is activated by binding to the NF-κBVIAA1.3-KBP fragment located at the upstream transcription initiation site of the human NLRP3 gene [19]. Studies have shown that adenosine triphosphate, reactive oxygen species, cholesterol crystals, oxidized low density lipoprotein, LPS and other cholesterol crystal factors can activate NLRP3 inflammasome [15, 33]. Lipopolysaccharide (LPS) is the main outer membrane component of Gram-negative bacteria, and it has been suggested that LPS from bacteria may be the source of the inflammatory response observed in atherosclerosis [34, 35]. In vivo experiments found that inflammatory factors were abnormally expressed in the aorta of AS model mice, so we verified in vitro whether IRN can reduce inflammation. In this study, we demonstrated that LPS induces high expression of NLRP3, NF-κB, Caspas-1, and IL-18 in HUVECs cells and RAW264.7 cells, suggesting that LPS can induce activation of NF-κB/NLRP3 inflammatory pathway (Figs. 2 and 3). LPS significantly inhibited cell migration, indicating that LPS reduced cell activity (Figs. 4 and 5).

IRN has been found to have therapeutic effects on cardiovascular and central nervous system diseases such as depression, Parkinson’s disease, Alzheimer’s disease and hypertension through mechanisms including antioxidant, anti-inflammatory and neuroregulatory activities [36, 37]. In this study, IRN treatment inhibited the activation of NF-κB/NLRP3 inflammatory pathway induced by LPS in HUVECs cells and RAW264.7 cells, and down-regulated the expression of NF-κB, NLRP3, Caspas-1 and IL-18 (Figs. 2 and 3), suggesting that IRN has an anti-inflammatory effect on NF-κB/NLRP3 pathway. In migration and scratch experiments, IRN promoted the migration ability of HUVECs (Fig. 4) and RAW264.7 (Fig. 5), indicating that IRN could protect cell activity. The results showed that IRN can protect the activity of HUVECs, thus reducing the damage of HUVECs caused by lipid metabolism disorder, inhibiting the inflammatory response induced by damage of HUVECs, and preventing the generation of atherosclerosis from the perspective of inflammation initiation. On the other hand, IRN can also protect the activity of RAW264.7, reduce the sensitivity of RAW264.7 to lipids and reduce the phagocytosis of lipids, thus reducing the formation of foam cells and ultimately reducing the generation of plaques.

In conclusion, IRN inhibits the inflammatory response of endothelial cells and macrophages induced by LPS, and its anti-inflammatory effect is related to the regulation of NF-κB /NLRP3 pathway. In addition, IRN can enhance cell viability. The results of this study provide sufficient scientific basis for the study of effective methods to inhibit the inflammatory response of AS.

## Supplementary Information

**Additional file 1.**

**Additional file 2.**

**Additional file 3.**

**Additional file 4.**

**Additional file 5.**

**Additional file 6.**

**Additional file 7.**

## Data Availability

The data and materials supporting this study are available with the Supplementary Information.
