# Supplementary material for: Isorhynchophylline inhibits inflammatory responses in endothelial cells and macrophages through the NF-κB/NLRP3 signaling pathway
Source: BMC Complement Med Ther. 2023 Mar 11;23:80. doi: 10.1186/s12906-023-03902-3 (PMC10007741; doi:10.1186/s12906-023-03902-3)

Original Figure 1E.

1. NLRP3


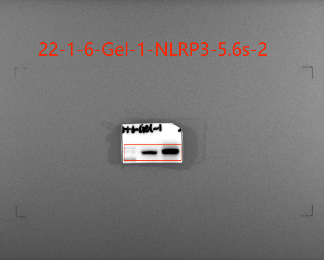


1. NF-κB


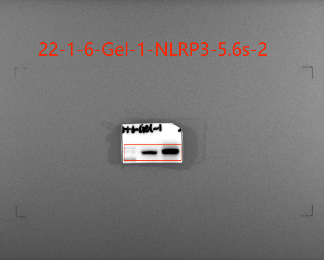


1. Caspase-1


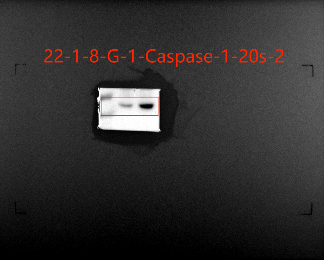


1. IL-18


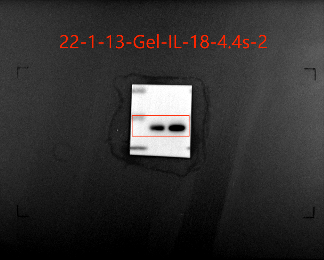


1. Actin


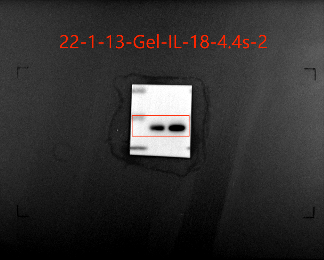


Original Figure 2A.

1. NLRP3


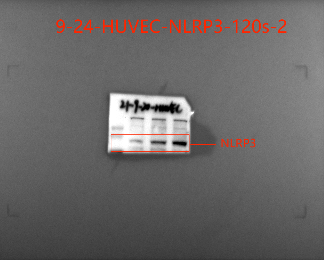


1. NF-κB


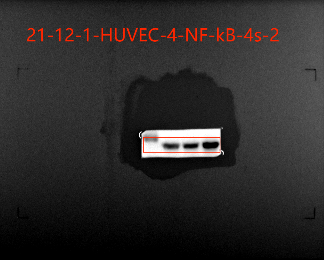


1. Caspase-1


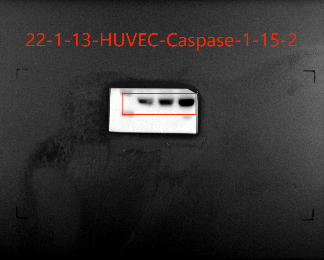


1. IL-18


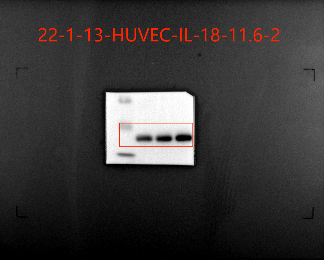


1. Actin


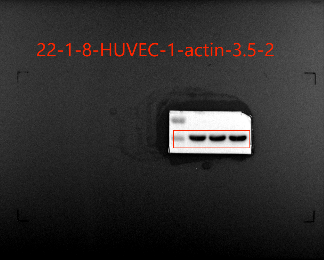


Original Figure 3A.

1. NLRP3


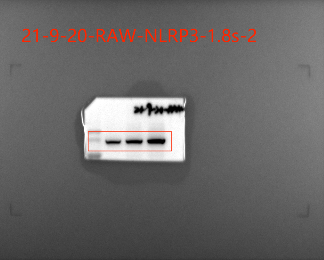


1. NF-κB


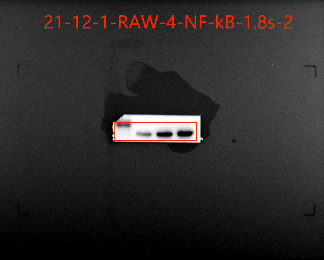


1. Caspase-1


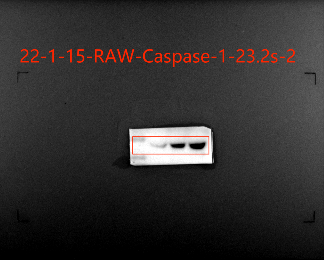


1. IL-18


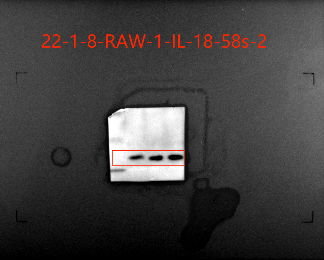


1. Actin


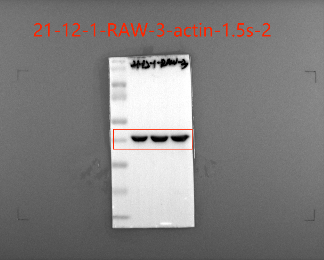

Supplement: Supplementary file 1 — Additional file 1. [file 12906_2023_3902_MOESM1_ESM.docx]
