# Supplementary material for: Isorhynchophylline inhibits inflammatory responses in endothelial cells and macrophages through the NF-κB/NLRP3 signaling pathway
Source: BMC Complement Med Ther. 2023 Mar 11;23:80. doi: 10.1186/s12906-023-03902-3 (PMC10007741; doi:10.1186/s12906-023-03902-3)

Western Blot: The one marked in red is the original image in Figure, the other two are the result of repeating it twice.

1.HUVEC(They were divided into control group, administration group and model

group.)

1. NLRP3 (The name 21-9-24-HUVEC-NLRP3-120s-2 and filled in red is the original image of Fig. 2A.)


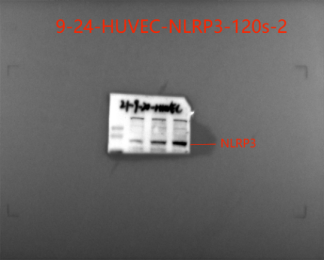

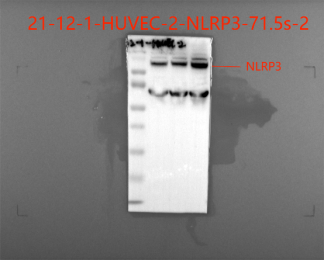

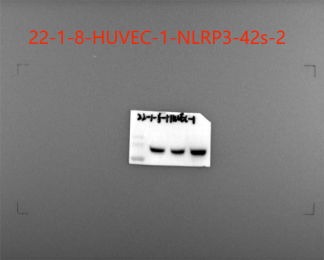


1. NF-κB (The name 21-12-1-HUVEC-4-NF-κB-4s-2 and filled in red is the original image of Fig. 2A.)


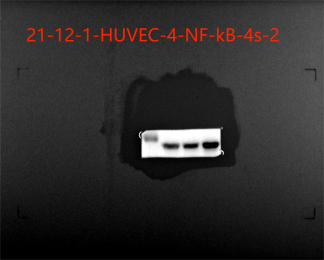

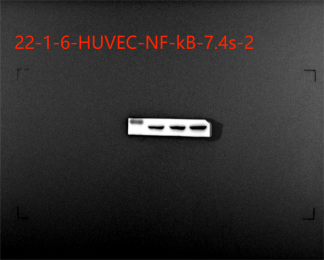

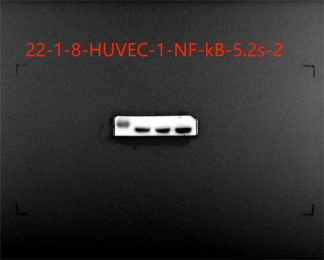


1. Caspase-1 (The name 22-1-13-HUVEC-Caspase-1-15s-2 and filled in red is the original image of Fig. 2A.)


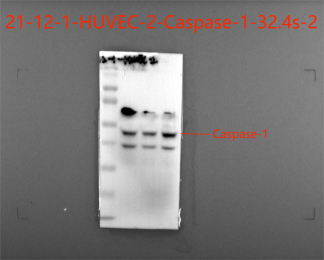

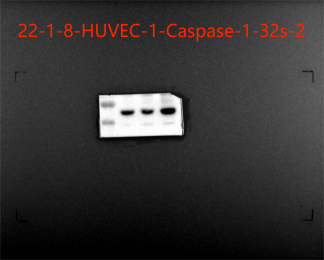

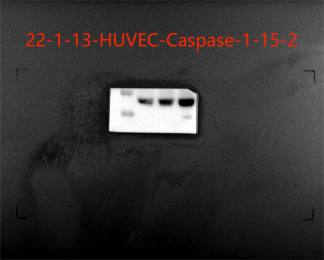


1. IL-18 (The name 22-1-13-HUVEC-IL-18-11.6s-2 and filled in red is the original image of Fig. 2A.)


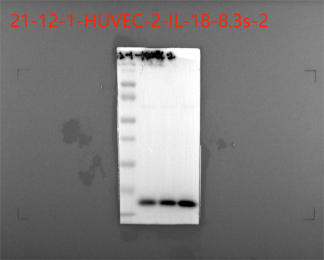

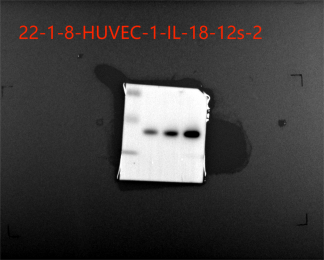

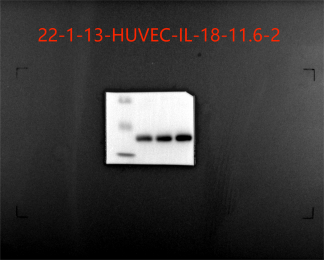


1. Actin (The name 22-1-8-HUVEC-1-actin-3.5s-2 and filled in red is the original image of Fig. 2A. The others are internal references corresponding to different target proteins.)


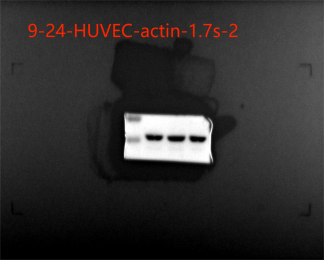

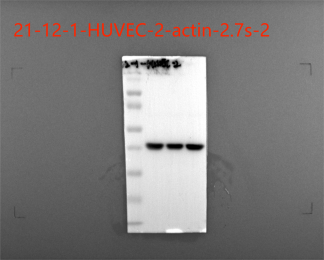

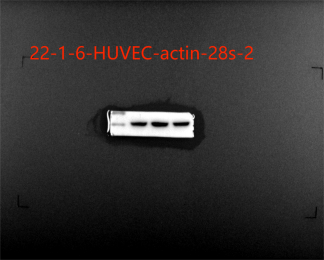

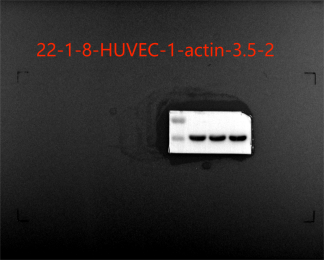

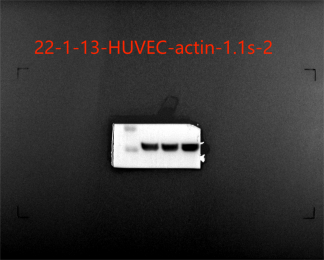


2.RAW264.7(They were divided into control group, administration group and model

group.)

1. NLRP3 (The name 21-9-20-RAW-NLRP3-1.8s-2 and filled in red is the original image of Fig. 3A.)


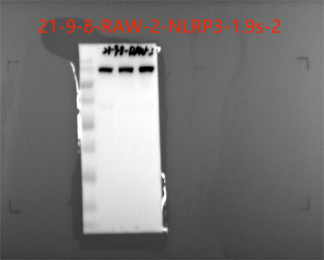

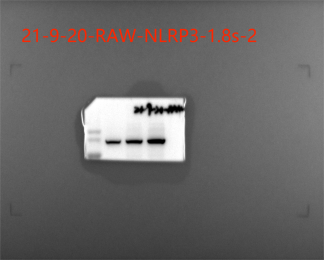

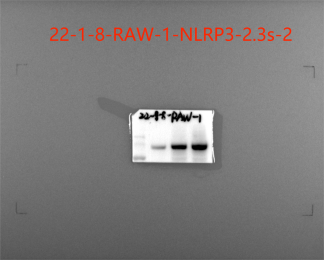


1. NF-κB (The name 21-12-1-RAW-4-NF-κB-1.8s-2 and filled in red is the original image of Fig. 3A.)


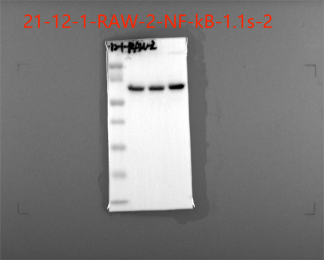

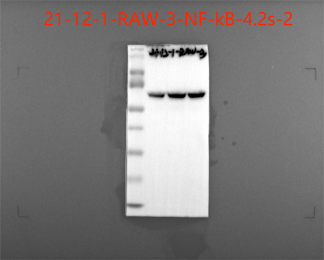

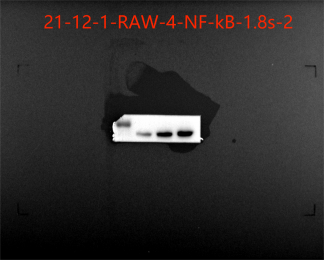


1. Caspase-1 (The name 22-1-15-RAW-Caspase-1-23.2s-2 and filled in red is the original image of Fig. 3A.)


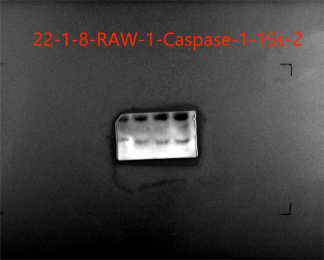

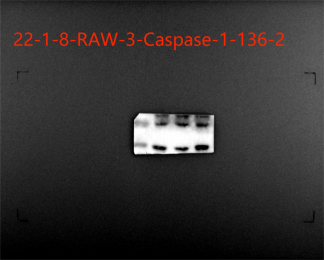

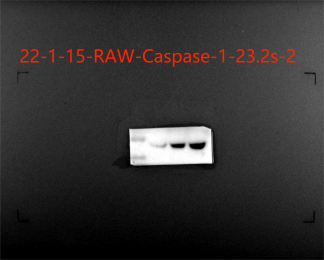


1. IL-18 (The name 22-1-8-RAW-1-IL-18-58s-2 and filled in red is the original image of Fig. 3A.)


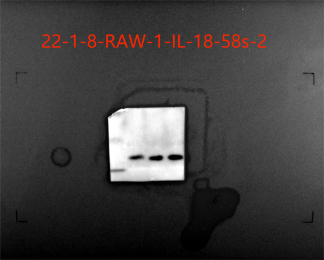

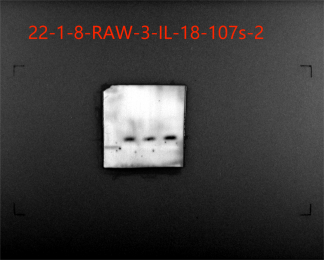

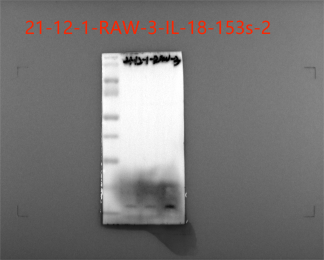


1. Actin (The name 22-12-1-RAW-3-actin-1.5s-2 and filled in red is the original image of Fig. 3A. he others are internal references corresponding to different target proteins.)


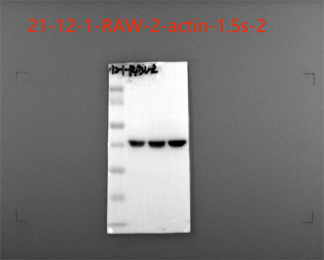

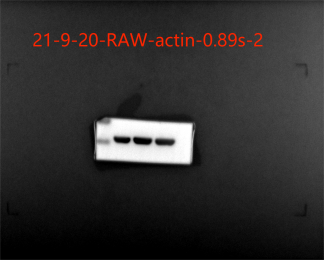

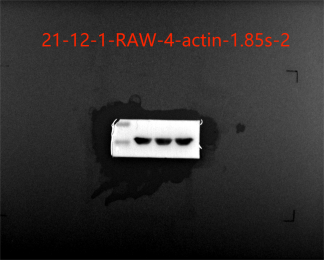

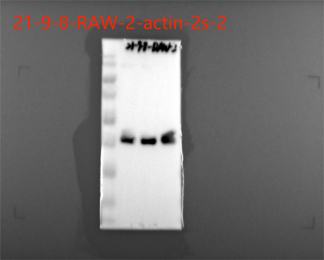

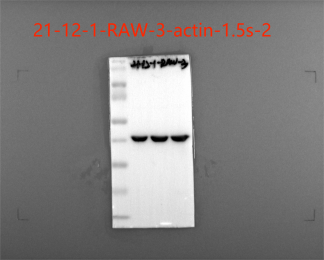

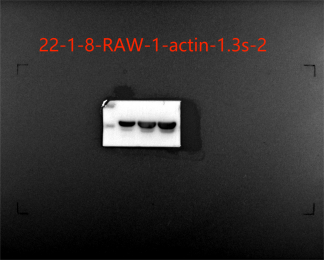

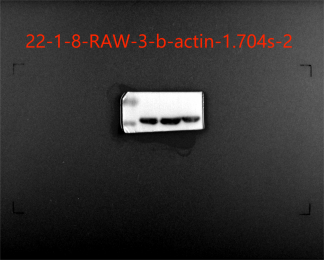

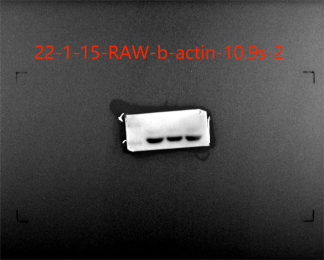


1. Animal(They were divided into control group and model group.)
2. NLRP3 (The name 22-1-6-Gel-1-NLRP3-5.6s-2 and filled in red is the original image of Fig. 1E.)


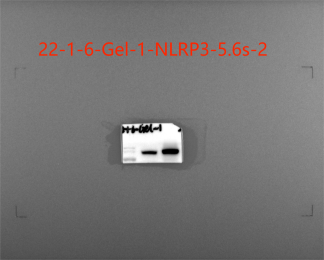

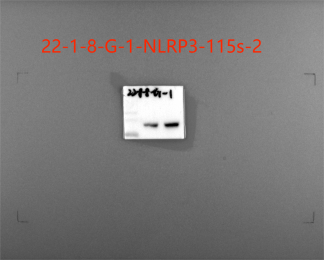

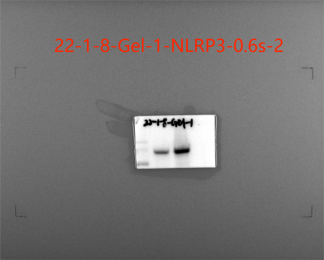


1. NF-κB (The name 22-1-8-Gel-1-NF-κB-26.5s-2 and filled in red is the original drawing of figure 1E.)


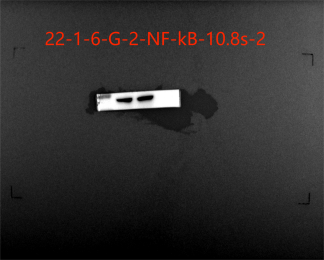

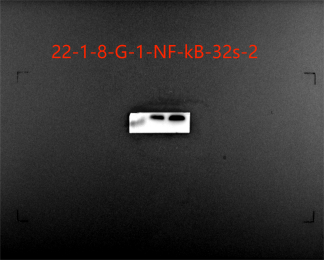

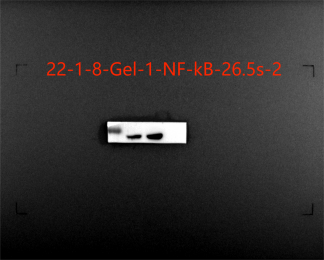


1. Caspase-1 (The name 22-1-8-G-1-Caspase-1-20s-2 and filled in red is the original drawing of figure 1E.)


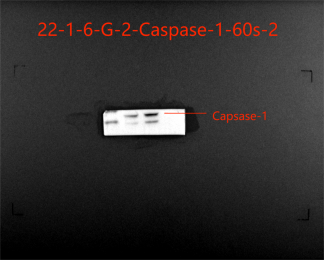

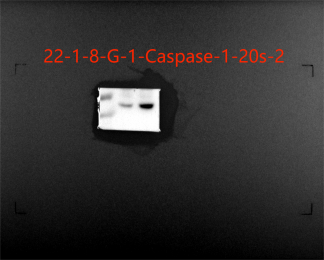

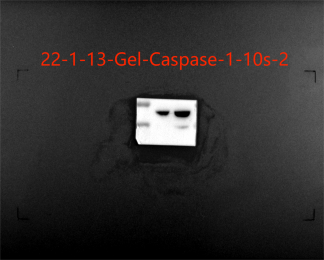


1. IL-18 (The name 22-1-13-Gel-IL-18-4.4s-2 and filled in red is the original drawing of figure 1E.)


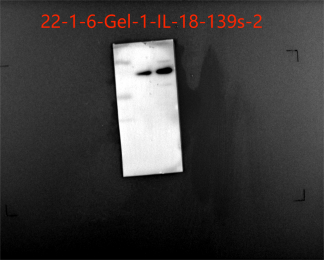

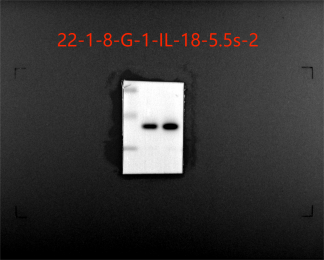

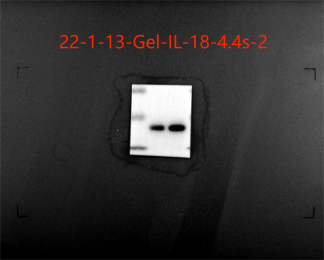


1. actin (The name 22-1-8-Gel-1-actin-6.3s-2 and filled in red is the original drawing of figure 1E. The others are internal references corresponding to different target proteins.)


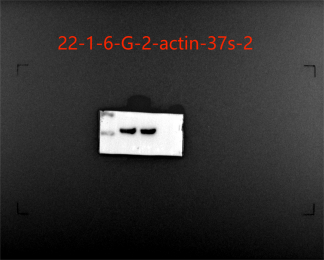

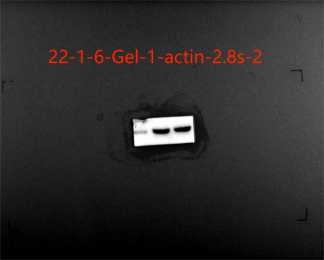

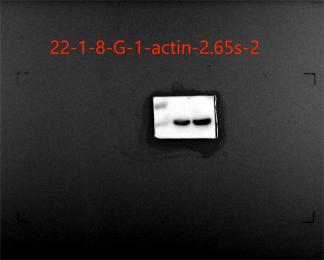

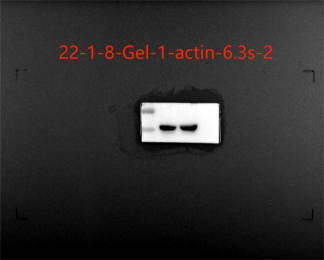

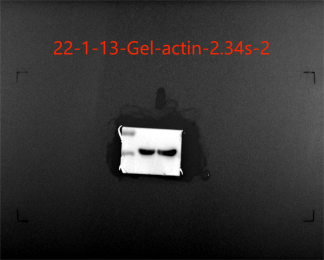

Supplement: Supplementary file 7 — Additional file 7. [file 12906_2023_3902_MOESM7_ESM.docx]
